# The Use of Chemical Control Within Coercive Controlling Intimate Partner Violence and Abuse

**DOI:** 10.1177/10778012231197579

**Published:** 2023-09-04

**Authors:** Sarah-Jane Walker, Marianne Hester, Elizabeth McCarthy

**Affiliations:** 1 1980 University of Bristol, Bristol, UK; 2 101845 Women's Aid Federation of England, Bristol, UK

**Keywords:** coercive control, chemical control, domestic abuse, intimate partner violence

## Abstract

This paper explores the use of chemical control by perpetrators as part of coercive controlling intimate partner violence and abuse, defined as the nonconsenting use of prescribed and nonprescribed medication (including vaccines), and/or other substances to coerce or control, reducing the victim-survivor's capacity for independence, freedom, and health. Based on testimonies of 37 victims-survivors and nine domestic abuse practitioners in the UK we identify varying tactics used to chemically coerce and control, deepening our understanding about the continually changing forms of domestic violence and abuse and enhancing the potential for a more robust response through better informed policy and practice.

## Introduction

Coercive control within intimate relationships refers to an on-going pattern of behaviors which, taken together, serve to dominate, undermine, and restrict the freedom of an individual (Stark, 2007). In 2015 in England and Wales, the offence of controlling or coercive behavior in intimate or familial relationships (Serious Crime Act 2015; s.76), was implemented to close an important gap in the law by including patterns of controlling or coercive behavior, including nonphysical and physical abuse, during an on-going relationship between intimate partners or family members. Victim-survivors of intimate partner violence and abuse (IPVA) have, for many years, described the on-going patterns of abusive behaviors used against them by their, usually male (ONS, 2022) partners, involving a range of behaviors and varying combination of types of abuse including psychological, physical, sexual, financial, emotional and threats as well as stalking and harassment—that the perpetrator uses to coerce them and control their lives. However, in terms of the criminal justice response, there has long been an emphasis on physical violence as domestic abuse-related offences (HMIC, 2014) with police officers still largely responding to individual “incidents” of IPVA. This focus on incidents of physical violence overlooks the many other coercive or controlling behaviors that perpetrators may use, which undermine the selfconfidence, selfesteem, and selfworth of victims-survivors over time, but may not be obvious to witnesses, police officers, doctors, lawyers, judges, juries, nor by the victim-survivor themselves (Williamson, 2000). Accounts from victim-survivors often reveal how it is these nonphysical aspects of abuse that have the greatest impact and are more difficult to deal with (Kelly et al., 2014; Stark, 2012; Williamson, 2010).

To date, research suggests criminal justice professionals may still be missing key opportunities for identifying patterned abuse and that the coercive control offence is not being used to its full potential (Barlow et al., 2020; Brennan & Myhill, 2022). A qualitative review of 18 cases prosecuted under s.76 found that the law on coercive control was being correctly applied to historical patterns of abuse that included multiple elements of coercion and control; that prosecutors were tapping repetitive physical and sexual abuse to show the “serious effect” required by the law; and that the vast majority of convicted offenders have received significant time in prison, up to a maximum of 6.6 years (Stark and Hester (2019). None the less, as the authors outline, it is too soon to determine whether the new offence will lead to promised improvements. While the number arrested under the offence is increasing, by 2019 fewer than 700 cases had been prosecuted under s.76 in England since its inception. Brennan and Myhill (2022), in a wider analysis of coercive control cases being processed by the criminal justice system in England and Wales by 2019, suggest that despite increases in reporting and arrests by the police the prosecution of such cases was even fewer than for domestic abuse crimes in general.

While the current definition of coercive control covers many behaviors this is by no means exhaustive. Also, perpetrators may change the forms of abuse they use over time and within different contexts. We were commissioned by the Oak Foundation to provide new knowledge and understanding about possible forms of coercive control that have not previously been dealt with to any extent. This included the potential use of “chemical restraint” by perpetrators in IPVA. However, while the (mis)use of substances such as prescribed medication can be used by domestic abuse perpetrators to psychologically, emotionally, or physically restrain/ constrain victims-survivors, it is important to distinguish this from the existing concept of chemical restraint (CR) that is applied within health care or institutional settings. The concept of CR in these settings refers to the use of interventions/ practices that restrict the rights or freedom of movement of an individual seemingly to prevent harm and although based on the fundamental guiding principles of safety, care, and well-being of individuals (Preisz & Preisz, 2020) is much critiqued as a form of restrictive practice (Edwards et al., 2020). Our research aimed to explore how IPVA perpetrators may be using similar “chemical restraint” against their victims. As we show below, our work has led us to use the term “chemical control” to better describe how domestic abuse perpetrators use chemical substances as part of their coercive control.

### Chemical Restraint: Previous Knowledge

Defined as the “forced (non-consenting) administration of medications to manage uncontrolled aggression, anxiety, or violence in people who are likely to cause harm to themselves or others” (Muir-Cochrane et al., 2020a), the concept of CR typically refers to drugs that are used for discipline or convenience instead of medical purposes and most often used to sedate, pacify and control behavior in emergency situations, for example, aggressive patients within health settings or offenders within criminal justice institutions. The existing literature addressing CR is mostly published in international gerontology or health and social welfare journals and largely related to its use within state or institutional settings, that is, in the public sphere as opposed to the private or domestic sphere. It covers elder abuse and neglect (e.g., Machado et al., 2001) and related debates around the quality of residential care for elderly adults including those cognitively impaired (e.g., Kaskie et al., 2015; Ralph & Espinet, 2019). It also addresses the use of CR to manage behavior within child mental health settings (e.g., Marzullo, 2014; Montreuil et al., 2020; Preisz & Preisz, 2020; Sorrentino, 2004); on institutionalized adolescents (e.g., Crespi, 1990); on adults with mental health disorders including psychiatric patients (e.g., Lee et al., 2003; Muir-Cochrane et al., 2020b; Wynn, 2004); and on adults with physical and learning disabilities (e.g., Donley et al., 2012; Edwards et al., 2020; Gulland, 2016; McSherry & Tellez, 2016; Webber et al., 2011). The literature also includes chemical castration, used in some countries to control sexual deviance in offenders, and the indiscriminate use of powerful psychotropic drugs in prisons has also been documented, for example as a way to “treat” the perceived “deviance” of female inmates (e.g., Auerhahn & Dermody Leonard, 2000; Crépault & Kilty, 2017). Thus, much of the existing research addresses the state-sponsored use of CR to control certain groups within the population, for example, to correct deviant behavior of offenders or manage harmful/challenging behavior amongst patients with mental ill-health. This use of CR may be justified to prevent harm and is managed through governance of risks and legal frameworks surrounding duty of care, informed consent and surrogate decision-making (Preisz & Preisz, 2020). Nevertheless, it is increasingly recognized for having negative consequences for an individual's dignity and safety and affecting their human rights, leading to growing calls for its use to be minimized or withdrawn from practice altogether (e.g., Edwards et al., 2020; Muir-Cochrane et al., 2020a; Preisz & Preisz, 2020).

### Chemical Control Within Gender-Based Violence Literature

Previous research conducted by the authors indicated that perpetrators of domestic and sexual violence and abuse may use substances, including prescribed medication, as part of coercive controlling domestic abuse (Women’s Aid, 2019), including the withholding of HIV medication in the context of intimate partner abuse (Donovan & Hester, 2014) and the use of drugs (such as crystal methamphetamine, GHB or mephedrone) in the context of “chemsex” making some men more vulnerable to abuse (Hester et al., 2019). Still, the wider gender-based violence (GBV) literature addressing the issue of chemical control within IPVA is still somewhat limited. Where the over-administering or deliberate withholding of medication has been recognized as a form of abuse (e.g., Machado et al., 2001; SCIE, 2015) it has tended to be listed as a specific type of abuse (e.g., elder abuse) distinct from IPVA. For example, guidance for frontline practitioners who work with adults with care and support needs and who may be at risk of abuse or neglect (SCIE, 2015) lists the “misuse of medication (over-sedation)” as a form of physical abuse but differentiates this from the forms of IPVA listed elsewhere in the guidance. This is possibly due to later life experiences of IPVA being treated as a sub-set of abuse against older people, ignoring the gendered power of abuse and experiences of older women (e.g., McGarry et al., 2011) and resulting in a blurring of boundaries between elder abuse and domestic abuse (Scott et al., 2004). However, we do know that adults- particularly older adults with physical or cognitive disabilities—can be abused by an intimate partner who may also be their designated carer (e.g., Carthy & Holt, 2016; Knight & Hester, 2016) leading to a number of physical and mental health implications for older adults living with IPVA (e.g., Lazenbatt et al., 2013; McGarry et al., 2011; Stöckl & Penhale, 2014) and, for some, their subsequent use of prescription and nonprescription drugs as a way of coping with the associated trauma (Lazenbatt et al., 2013; Wuest et al., 2008).

The mis(use) of medication within IPVA has been associated with reproductive coercion or reproductive control (RC). First recognized in 2010 RC encompasses a range of behaviors that aim to interfere with or restrict reproductive autonomy and can involve pregnancy coercion, birth control sabotage, and controlling the outcome of a pregnancy (e.g., Alhusen et al., 2020; Fay & Yee, 2018; Grace et al., 2022; Hardesty & Ogolsky, 2020; Kovar, 2018; Thaller & Messing, 2016) and which disproportionately affects younger, marginalized women (Bagwell-Gray et al., 2021) and women experiencing physical violence (e.g., Katz et al., 2017). Furthermore, research does show a complex relationship between substance use and IPVA where both intoxication and withdrawal can preempt perpetration (Gilchrist et al., 2019) with research highlighting the effects of substance use on safety, frequency and severity of IPVA (e.g., Gadd et al., 2019; O'Brien et al., 2018). However, while existing evidence illustrates an undeniable and nuanced association between substance use and IPVA the focus of our research moves beyond the co-occurrence of domestic abuse and drug and alcohol misuse to further explore the use of prescribed medication/treatment, over-the-counter medicines and vaccines to coerce and control an intimate ex/partner. We know that perpetrators often use forms of abuse which make use of, and exploit, a victim-survivor's impairment, or condition, compounding the violence experienced (e.g., Macleod & Cosgrove, 1995; Magowan, 2004) and that people with disabilities may experience more extreme exercise of power, coercion and control than those without disabilities (Hague et al., 2008) including being denied access to vital medication (e.g., Hague et al., 2011; Nosek et al., 2001; Plummer & Findley, 2011). Research clearly shows disability/impairment can be used by a perpetrator and have a profound impact on the victim-survivor (e.g., Hague et al., 2008; Mays, 2006) however the specific use of medication and other chemicals by perpetrators to coerce and control within IPVA—and how this might manifest amongst victims-survivors with, and without, disabilities/impairments—remains under explored.

This paper reports on the findings of research conducted as part of a wider Oak Foundation project on coercive control, with the focus here specifically on use of prescribed medicines and other substances as a type of “chemical control” within the context of IPVA. The research was approved by the University of Bristol's Research Ethics Committee.

## Method

We used a qualitative approach that included three elements: (1) re-analysis of six interviews from the recently completed Justice, Inequality and Gender-Based Violence project^1^; (2) an online victim-survivor survey resulting in 27 detailed responses and four individual online interviews; (3) an online focus group and interview with nine IPVA sector professionals. Thus, the paper draws on the testimonies of 37 IPVA victims-survivors (survey = 27; interviews = 10) plus nine professionals who work with IPVA victims-survivors.

For the re-analysis, the six interview transcripts were drawn from a wider sample of 251 victim-survivors previously (2015–2018) interviewed about their experiences and perceptions of “justice” in relation to GBV (e.g., Gangoli et al., 2019). The 251 interviews were electronically searched using the search terms “medic”; “prescri”; “sedat”; “restrain”; “incapac”; “withh”; “chem”; “alcoh”; “drug”; “drink”; “cocaine”; “pill”; “tablet” to identify any victim-survivors who had specifically mentioned the (mis)use of prescribed medicines, vaccines or other substances as part of intimate partner abuse.

In July 2021 we designed and distributed a short online survey, in consultation with Women's Aid Federation of England (the national umbrella organization for IPVA support), seeking detailed input from adults (≥18 years, all genders, sexual orientations, nationalities, ethnicities, faiths or no faith, regardless of immigration status) who had experienced coercive control where an intimate partner or ex-partner had used prescribed medicines and treatment (including vaccinations and birth control) and/or other drugs (including illegal drugs) as part of their abuse. Questions were asked about experiences and impact (see Table 1 for questions linked to the use of medication and other substances as part of intimate partner abuse). The survey was advertised via Women's Aid's existing Survivor Forum with a message on the Forum providing information about the research and consent and asking for participants to take part. The survey was also disseminated via existing GBV networks such as VAWGRN; British Sociological Association VAWG network and sent to local and national IPVA organizations requesting they distribute via their networks (including Respect Men's Advice Line, Galop and Imkaan). Respondents were given the option of either completing the anonymous qualitative survey online or, if they preferred, taking part in an in-depth online interview (via Zoom) with a researcher. The survey yielded 27 complete online responses and online interviews were conducted with an additional four participants using the survey questions. The interviews were recorded and stored directly onto the password protected university server. Notes were also taken at interview, anonymized and stored on the university's secure server.

**Table 1.** Victim-survivor experiences (*n* = 37).

	*N*	%
*Partner or ex-partner deliberately withheld essential medication or treatment (including vaccines) from them and/or their child(ren)*	11	30
*Partner or ex-partner controlled, or tried to control, their access to medication/treatment, for example, by withholding the money or other means necessary for them to access it*	9	24
*Partner or ex-partner used, or tried to use, other substances (apart from prescribed medication), including illegal drugs and/or alcohol, to make them do something they did not want to do*	19	51
*Partner or ex-partner had controlled, or tried to control, their contraception (e.g., tried to get them pregnant, or tried to get pregnant, against their wishes by sabotaging birth control method(s)*	9	24
*Partner or ex-partner had interfered, or tried to interfere, with them getting a COVID-19 vaccination, or had forced them to do so*	5^a^	16

a Excludes 6 respondents from the Justice project which was conducted before the 2020 COVID-19 pandemic.

We conducted one online focus group (involving eight participants) and one online interview (both via secure Zoom) with professionals from specialist NGOs (recruited through emails sent directly to these organizations) including services run by and for Black and minoritized victims-survivors.

The qualitative survey and interview data were analyzed using thematic coding via repeated reading of the data (Ritchie et al., 2013) to link and build on the themes identified in the earlier stages. Pseudonyms are used throughout this paper to protect the identify of participants.

## Findings

The vast majority of victims-survivors in our sample were women (who identified as heterosexual, bisexual or pan sexual—see Table 2) with male perpetrators. One victim-survivor was a man with a female perpetrator. The majority (81%) reported having a physical or mental illness/condition and of these 96% had illnesses/conditions lasting, or expected to last, 12 months or more (which may or may not have involved the use of medication). Just over two thirds of respondents (68%) believed the perpetrator had used their long-term illnesses/conditions against them as part of their abuse with the majority (86%) reporting their long-standing illness/condition was a direct result of the abuse experienced.

**Table 2.** The Victims-Survivors: Sample Overview (*n* = 37).

	*n*	%
Woman	36	97
Man	1	3
18–24 years*^a^*	6	18^b^
25–34 years^a^	17	50^b^
35–44 years^a^	4	12^b^
45–54 years^a^	7	21^b^
*Heterosexual*	32	91^c^
*Bisexual*	2	6^c^
*Pansexual*	1	3^c^
*Asian/Asian British: Pakistani*	4	11
*Mixed/Multiple: White and Asian*	1	3
*Mixed/Multiple: White & Black Caribbean*	2	5
*White: English/Welsh/Scottish/Northern Irish/British*	27	73
*White: Irish*	1	3
*White: other European*	1	3
*Other*	1	3
*Has physical or mental illnesses/conditions*	30	81

a Age at time of abuse.

b Excludes missing data (*n* = 3).

c Excludes missing data (*n* = 2).

### The Use of Prescribed Medication/Treatment to Coerce and Control

Based on key themes emerging from the existing literature and re-analysis of our previous research data, we asked participants if they had specifically experienced the withholding, control or attempted control of prescribed medication or treatment (including vaccines and contraceptives, see Table 1). Participants were then asked to describe their experiences in their own words.

Analysis of the survey and interview data revealed numerous ways in which perpetrators used their partner's prescribed medication/treatment (and other substances) as part of their abuse, with devastating impacts for victims-survivors (and their children), notably a reduced ability to manage their own physical and/or mental health. The various perpetrator tactics are discussed under the following key themes: withholding and administering; sabotage and gaslighting; sexual coercion and exploitation; RC; the procurement of drugs for own use; and branding*.*

### Withholding and Administering: Two Sides of the Same Coin

Nearly one in three (30%) victims-survivors reported that a partner or ex-partner had deliberately withheld essential medication or treatment (including vaccines) from them and/or their child(ren). One, Beatrice, reported that her abusive ex-partner would deliberately withhold her pain medication on a regular basis with devastating effects on both her physical and mental health reporting “my pain levels became so high I was regularly hospitalized or bed-ridden, making me feel desperately isolated and unable to cope with daily life leaving me feeling suicidal*.*” Another, Elaine, was diagnosed with a life-threatening illness in her early twenties, needing several major surgeries and 24-hour care during her recovery. It was at this point her (now ex) partner insisted they secretly marry and move house. Once fully dependent on her partner, Elaine said he used her need for medication and care to increase his control stating “we moved an hour and a half away from everybody that I needed. When I look back [that's] when it started. He’d withhold medicine and he’d choose who was allowed to come up to the house, I had to be bathed, I couldn’t physically do anything myself, he’d decide when that happened or if that happened.”

Many of the professionals interviewed were familiar with the notion of perpetrators withholding victim-survivor's medication as a tactic of abuse. One recalled working with a victim-survivor who was diabetic and whose partner would deliberately withhold her insulin unless she complied with his demands, while another suggested they had seen a large increase in referrals from gay or bisexual men and a small number of trans+ women where the issue of chemicals had had a huge impact in intimate partner relationships, including the use of prescribed medication,

For example, withholding (including access to) medication to trans+ people [or] HIV/AIDS medication—we’re not seeing these in any significant numbers but are aware that for some people this has been an issue … it might be withholding medication or destroying clothes—we’ve definitely had trans people disclose those things to us as part of a wider issue around coercion and control. (Practitioner-09)

In cases like those of Beatrice and Elaine, the withholding of vital medication was a direct and overt tactic where the perpetrator did not even try to conceal their (mis)use of the victim-survivor's medication, with potentially serious physical impacts for that victim-survivor. However, respondents also revealed that perpetrators can, and do, use essential medication in other, more *indirect* ways to control and abuse. Francis's ex-partner used various professionals (police, children's services, doctor) against her as part of his control and abuse and at the time of interview, she was in a protracted custody battle. She explained that as part of her ex-partner's attempts to control her, he deliberately withheld their daughter's medication,

I [was referred] to children's services because in his mission to prove that there was nothing wrong with our daughter he decided to stop her [] medication and she ended up on steroids because her [condition] deteriorated… it went to court and he's lied, it should never have gone to court, even the judges have said that… it's still in court… even though he’d stopped the medication, social services believed in the stereotypical mother trying to stop contact with father when I reported him for stopping her medication. I’ve just had a complaint upheld by an independent investigator; I’ve had something like 36 apologies from social services for their failings.

On the flipside of *withholding* necessary medication as a means of control, perpetrators were also reported to control via the *administration* of medication. Mandy, for example, described how her husband took it upon himself to dispense her medication for her, which she later recognized as a deliberate attempt to control her,

[he] has always dished out my prescribed meds, saying I was too weak to pop the foils etc. It used to suit him fine if I was “out of it”, [he] seemed to enjoy me being more needy i.e., sleepy or frail. [it made me feel] pathetic, unable to make decisions, that I had no control, just take whatever is given with NO hope of improvement if I don’t take them all.

Some of the more covert tactics used by perpetrators to weaken or incapacitate victims-survivors, included the “spiking” of food or drinks with medication and/or other substances. Beth, for example, described experiencing multiple forms of abuse from her ex-husband including physical violence, verbal, emotional and psychological abuse and control. In terms of the use of medication in particular, Beth suggested that despite having no concrete evidence she strongly suspected her husband (a senior health professional) not only of interfering with her birth control pills (ensuring she fell pregnant with all four of their children) but also of “drugging” her drinks. She explained that despite his constant verbal and emotional abuse towards her, every morning without fail he would take her a cup of tea,

In my stupid little world, I thought ‘oh he still cares about me, bringing me a cup of tea every morning’. But the only reason I can think of now was that there was something in [the tea]. I didn’t feel like I had the fight in me to confront him and I suspect it was some sort of calmer, you know, something to keep me sedated a little bit. Even though I was driving his kids around, which is actually quite scary.

One practitioner reported that the abusive partner of one victim-survivor they were supporting would grind up that victim-survivor's sleeping tablets and mix them into the sugar, in order to effectively drug her tea and coffee. When she was under the effects of the sleeping tablets the perpetrator would place her in sexual positions, photograph/ record her, and threaten to show the recordings to others if she ever left him, including to social services in order to sabotage her chances of gaining custody of her children. He also threatened to post the “compromising” photos of her in public. Having her drinks “spiked” in this way meant the victim-survivor would often present as if she was not “alert” when collecting her children from school and, as a result, social services eventually became involved. In Olivia's case, her ex-partner gave her strong opioid painkillers whilst telling her it was “no big deal.” Upon reflection she believes this was a deliberate strategy to manipulate her into dating him i.e., for his own convenience rather than hers,

I had no idea what [it] even was at the time and that it was extremely addictive. I ended up addicted to it for 2 years. I have had to stay on [drug treatment] for 13 years now to make sure I never take it again. It made me feel very manipulated. I just felt as if he preyed on my naivety as he tried to date me for a long time and I wouldn’t. I finally gave in and dated him and I just feel as if he gave me that and didn’t tell me how addictive it was on purpose as a way to be able to control me and keep me dating him.

### Sabotage and Gaslighting

While in many cases the tactics used by perpetrators were blatant and carried out in plain sight, perpetrators would also use prescribed medication to abuse in more furtive ways. Practitioners reported working with survivors whose abusive partners had deliberately disrupted their medication, including mental health medication, for example, by hiding the medication and then either denying it or blaming them for losing it. Tactics like this are liable to exacerbate the victim-survivor's existing mental health issues (Women’s Aid, 2019) yet while victims-survivors may possibly recognize situations like these as problematic, they might not necessarily recognize it as abuse until they are supported to understand what was happening to them, suggesting “*they don’t see it as abuse and are quite shocked to learn that it is domestic abuse*” (practitioner-02).

Debra described how her mental health had been negatively affected as a result of being in an abusive relationship and subsequently sought help from her doctor who prescribed her antidepressants,

[my partner] was very against this…he started to hide the tablets, basically from day one. When I asked where the tablets where he would pretend I was going mad by making out he hadn’t touched them…[I] found them in strange places…I recognise now that he didn’t obviously want me to start feeling better as he could control me better in the state I was in without them.

Continuing his abuse, Debra's partner successfully thwarted her efforts to feel better as his behavior meant she never took her mediation properly and eventually stopped altogether, “*I have never told anyone he restricted my access to the tablets and since then have never been back to my GP*.”

Victim-survivor narratives also revealed that some perpetrators controlled, or tried to control, the use of vaccines as part of their coercive controlling behaviors. One participant, Anna, reported that her partner would not “allow” their children to be vaccinated (against her wishes), while another, Ellen, also reported that her husband would consistently try to prevent her, and their children, from seeking medical help. She said he would become aggressive and verbally abusive if he ever found out they had sought or accessed help from any medical professionals (including dentists). While her children did receive some vaccinations (administered via school) any other medical appointments required would have to be arranged/ conducted without his knowledge or she would “suffer the consequences” of his extreme anger. Practitioners suggested that children in refuge are often not up to date with their essential vaccines, again because their mothers’ abusive partners had not “allowed” their children to be vaccinated.

Of those respondents surveyed/interviewed in 2021, almost one in six (16%) reported a partner or ex-partner interfering, or trying to interfere, with them receiving a COVID-19 vaccination, or said they had pressured them to do so, as part of a pattern of abuse. Again, the experiences described by the victims-survivors highlight the lack of control they felt they had over their own bodies and physical health, unable to make their own decisions when it came to being vaccinated against COVID-19. For example, Ingrid reported that her partner “*bullied me about getting the covid-19 vaccination. He was bullying me into booking it and making me feel very pressured*.” Another, Julie, experienced “*rage, silences, insults and belittling if [I] went against what he deemed best re wearing of face masks and vaccination*,” which made her feel “*like a child. Frustrated. Confused. Scared*.”

Conversely, while some respondents reported that their abusive partners would control their medication, either by withholding or sabotaging their access to, for example, antidepressants when they wanted to improve their mental health, others described how their abusive partners had convinced them to take medication that they did not actually need. Claire, for example, said “*my ex convinced me to go to the doctors as he convinced me I was off my head and that I needed medication and I ended up taking anti-depressants when the problem wasn’t really with me*.”

One participant, Anna, reported experiencing physical, emotional, sexual abuse (including unwanted sex with others while she was pregnant, controlling contraception, influencing pregnancy and termination decisions) over a period of 20 years. She explained that at one point her ex-husband had persuaded her own doctor to prescribe her with antidepressants despite her insisting she did not want to because she was breastfeeding at the time,

[my partner] told them I needed pills and the doctor put me on pills. He sat in on doctors’ appointments and spoke to the doctors for me … which I can’t believe looking back now that no doctors had a red [flag]. Because I’m educated and eloquent, I’m more than capable to speak for myself, and patient confidentiality … I mean it's just not normal … you know I’d say to the doctors ‘Oh I don’t want to go on medication cos I’m breast feeding’—he would convince the doctors to convince me to stop breast feeding so I could be on medication so he could control me more.

At the time of interview Anna was still taking those antidepressants, 12 years after she had first been prescribed them and seven years after she had separated from her husband, saying she finds it difficult to stop taking the medication. Others also recognized this as a tactic of control within domestically abusive relationships, for example, Paula said “*I know of people saying their partners have tried to get them to use prescription drugs. By making their partner anxious, fretful, crazy etc they are then taken to the doctors by the abuser*.” While Rachel reported, “*[my] ex tried to force codeine and diazepam on me so he could further gaslight me and live a separate life outside the house cheating on me and generally doing as he pleased … He also messed with my head to the point I ended up sectioned*.”

### Reproductive Coercion and Control

Almost a quarter (24%) of victims-survivors in our sample reported that their partner or ex-partner had controlled, or tried to control, their contraception, for example, tried to get them pregnant, or tried to get pregnant, against their wishes by sabotaging birth control method(s). Gail reported that her ex-partner had raped her which resulted in a pregnancy, saying “*the next day I wanted to get the morning after pill, he wouldn't let me. He also used to discard my contraception so I couldn’t take it. I wasn’t allowed to go to the doctors without him*.”

One practitioner supported a woman whose ex-partner would remove her contraceptive pill from the package,

She already had children through sex she hadn’t consented to. She wasn’t allowed to have a termination and forced to have sex. If she had sex [she] would worry until her next period. [She was] constantly frightened that she would be pregnant again. [It was] another threat to hold over her. (Practitioner-01)

In fact, practitioners mentioned several cases where a victim-survivor's pill had been taken out of the package by their abusive partner suggesting his would often lead to the woman questioning herself, “Did I take it or not?” One recalled supporting a woman whose partner would “not allow” her to have an intrauterine device (IUD) fitted because having such a device would mean contraception was then out of his direct control.

### Sexual Coercion and Exploitation

Over half (55%) the participants said that a partner or ex-partner had used, or tried to use, other substances apart from prescribed medication, including illegal drugs and/or alcohol, in order to make them do something they did not want to do. Harriet was unwell for several years with a (undiagnosed at the time) painful condition, which was impacting her mobility and functionality,

My ex-partner knew I was on heavy pain meds (opiates) and courses of IV steroids, but he would coerce me into drinking alcohol with him under threat each night when he came home … He would also add alcohol to my coffee without my consent. The mixture was dangerous and at times led me to be very dizzy, disoriented and on more than one occasion lose consciousness. It made me feel scared and even less in control of my own life then I already felt.

Participants described various ways that their abusive partners would use alcohol and/or other drugs in order to sexually coerce, abuse and exploit. Lisa disclosed,

[perp] often used alcohol as a way to gaslight and ramp up abuse as my memory was impaired…He also sexually assaulted me when I was drunk and he was sober, I believe, because he was angry with me. [I felt] guilty. I believed that I was the one in the wrong. [he] made me feel like I was losing control; like I was a mess.

Natalie reported that her partner is prescribed a version of Viagra via his GP saying “*this tablet is a living nightmare for me!! Because I have to ‘allow’ him to have sex with me as when using these tablets it all has to be ‘planned’ … [I’m] absolutely sick of it*.”

Carol experienced emotional and psychological coercive control throughout her 20-year relationship with her husband. As part of his abuse her husband would force Carol to have sex with other men for money, keeping the money “earned” for himself. He would also take explicit photos of her and post them online to earn money, plying her with alcohol to reduce her resistance and make her easier to control,

He used to take horrid pictures of everything down below. It got to the point where he used to post stuff on an escort website, post pictures of me … not my face … as a way of creating more income…there was pictures of me on there, and men had to pay to get their kicks, and he would get that money. (…) Made me have a threesome, … got me drunk … I don’t know if he’d given me anything, but yeah to force me to do things that I didn’t want to.

Danielle disclosed similar experiences of sexual coercion at the hands of her partner,

When I arrived home drugs and alcohol would be already bought and on the table for me to take even when I wanted to abstain. Sometimes his friend would be involved, and I would be coerced to take the drugs and drink and have group sex. My husband liked to watch his friend having sex with me and drugs were an integral part of how this came about.

### Procuring Drugs for Own Use

One respondent, Joanne, experienced emotional, verbal, and financial abuse and coercive controlling behavior from her male partner (at the time of interview, she was experiencing on-going, post-separation stalking and harassment from him including threats to harm). Joanne has a long-term condition meaning she needs to take strong opioid painkillers daily to manage her pain. However, she explained that her partner, who was also her designated carer, would either steal and sell her medication to finance his drug addiction or he would wait for her medication to take effect and steal her money while she slept. He would persistently harass her for money and/or her medication, sometimes for up to 10 hours straight until she relented and gave him what he demanded. As a result of her partner's behaviors Joanne was often left in pain and/or without money to buy alternative painkillers (as well as electricity and food) sometimes for days. Joanne now also suffers with depression and anxiety as a direct result of the abuse she experienced (and still is experiencing) from her ex-partner.

A similar experience was recalled by another respondent, Kelly, who reported that her husband would use her prescription for opioids (which she needed for an on-going painful condition) for his own personal use, forcing her to order more or using her details himself to order prescription strength pain medication from the internet for his own use. Without medication specifically prescribed for her Kelly was forced to suffer in pain. The testimonies of both Joanne and Kelly mirror those of other victims-survivors whose perpetrators are also perceived to be their “carers,” thus making them vulnerable to control and economic abuse which includes being denied access to funds for prescriptions (Women’s Aid, 2019).

### Branding

Our research highlighted a range of perpetrator tactics involving the use of prescribed medication and other substances to abuse, undermine and control victims-survivors. Although the primary focus of this research was on the use of prescribed medicines/treatment as a method of coercive control respondents also reported other forms of abuse involving the use of chemicals. Practitioners reported cases of perpetrators deliberately putting substances such as pesticide sprays or super glue into a victim-survivor's eyes or hair, weaponizing such “ordinary” household substances to cause serious harm to the victim-survivor. One practitioner recalled a case where the perpetrator would pick out his partner's skin with tweezers and rub substances such as chilli powder or cleaning products into the wound (he had done this to multiple female partners). This would burn, leaving the victim-survivor with permanent scars, adding emotional trauma to the physical pain as the resulting scars acted a permanent reminder of their control and abuse.

## Discussion

The concept of coercive control encourages a deeper appreciation of the hidden and intrusive consequences of “dimensions of partner abuse that have gone largely unnoticed and that are not normally associated with assault” (Stark, 2007) such as, for example, psychological abuse, intimidation and isolation (Walklate & Fitz-Gibbon, 2019). As shown, perpetrators can, and do, use prescribed and nonprescribed medication, sometimes in combination with other substances to coerce and control their partners, using various chemicals as a form of nonviolent tactic that can be more devastating and salient to victims than physical violence (Stark, 2012). The survivor testimonies presented in this paper demonstrate how medication can be “weaponized” by perpetrators as part of abuse, to either *physically* constrain, for example, by ensuring the victim-survivor was sedated, pacified or totally dependent on their abuser, or to *psychologically* control, for example, by convincing the victim-survivor that they were mentally ill when they were not, so they would take medication they did not need. Perpetrators use such chemicals to regulate and/or isolate the victim-survivor (reflected in the experiences of Beatrice, Elaine, Debra, Mandy, and Olivia) and compel them to do as the perpetrator wishes, as well as to exploit and financially abuse (as in the experiences of Joanne and Kelly) where the perpetrator takes advantage of the victim-survivor's need for prescribed medication for their own personal gain, and without due care for any subsequent health consequences. Victim-survivor testimonies reveal how these diverse tactics work to reduce the victim-survivor's personal capacity for resistance and independence by taking away their independent judgment or thought (Stark, 2007) as well as their ability to function physically (as in Beatrice's experience).

Our research also exposes the potential for perpetrators to (mis)use medication and other substances to discredit them, for example in their role as a mother, often with the underlying threat of sabotaging custody of their children. We provide further evidence that children can be targets of coercive control both alongside and independently of their mother (Callaghan et al., 2018; Katz, 2016) with the deliberate withholding of Francis's daughter's medication, which can be seen as a direct form of physical abuse towards the child and a simultaneously indirect way to emotionally “restrain”/control Francis post-separation, rendering their daughter a “secondary victim” as her health care needs were used by her father to “score points” against her mother. Such abusive behavior goes beyond a child “witnessing” or being “exposed” to IPVA, illustrating how children can be used as pawns or instruments in perpetrators’ control strategies and is an example of the known overlap of domestic abuse with coercive control and child maltreatment (Stark & Hester, 2019). As in Francis's experience (and others we heard about), the “spiking” of victims-survivors’ food/drinks was seen as a deliberate strategy to undermine their integrity as a mother capable of caring for her children, endeavoring to ruin the victim-survivor's reputation through shaming and/or discrediting her in the eyes of the authorities (e.g., social services) and the wider community.

The testimonies of Carol and Danielle, presented earlier, demonstrate the (mis)use of medication and/or other substances to sexually coerce and abuse. We know that sexual abuse and violence is a frequent part of women's experiences of domestic abuse, and it is the prominence of sexual abuse that is a key difference between women's and men's typical experiences of intimate partner abuse (Hester & Lilley, 2018). The experiences presented in this paper support existing research evidence on sexual coercion (Williamson, 2014) which reports alarmingly high rates of women being coerced or pressured into sexual experiences (Women’s Aid et al., 2021). They also highlight some of the more under-researched elements of GBV, such as spiking in the context of IPVA, for example, to sedate or confuse an intimate partner or coerce them into unwanted sex with third parties (Matolcsi, 2020) which is less recognized than, for example, drink spiking and sexual assault in the context of the night-time economy. It can be argued that using medication and/or other substances to sexually coerce (experienced by Lisa, Natalie, Carol and Danielle), not only limits the individual victim-survivor's autonomous decision-making about when, where and who they have sex with but, at the wider (societal) level, works to perpetuate sexual inequality between women and men by continuing to propagate the notion that women are objects for men's use and pleasure (McKinnon as cited in Stark, 2007).

A key tactic of abuse reported by participants was the sabotage of their chosen contraception method (e.g., the experience of Gail) reflecting the known association between IPVA and reproductive coercion/control which is often utilized to maintain power and control in a relationship (Kovar, 2018) and which commonly co-occurs with other types of abuse, particularly sexual violence in relationships (Bagwell-Gray et al., 2021). Almost a quarter of our participants reported that their partner/ex-partner had tried to get them pregnant against their wishes by sabotaging birth control, higher than existing prevalence estimates of 8% to 16% (Kovar, 2018). The high prevalence of RC amongst victims-survivors was substantiated by the IPVA practitioners interviewed for the study and adds to the growing knowledge base around RC as a form of coercive controlling IPVA.

Echoing findings from elsewhere (Hester et al., 2021) victims-survivors tended to only recognize the (mis)use of medication and other substances as tactics of coercive control retrospectively and not whilst still in the abusive relationship. This reflects the insidious nature of coercive control, often difficult to recognize because the associated tactics often coalesce with normalized expectations of male and female behavior making it particularly hard to detect, and for women to articulate (Burman & Brooks-Hay, 2018; Stark, 2007). Many of the survivor narratives alluded to the long-recognized gendered nature of coercive control (Barlow et al., 2020; Stark, 2007; Williamson, 2010) which is reflected in the latest statistics which show 97% of those convicted for controlling or coercive behavior in England and Wales in the year ending December 2020 were male (ONS, 2021). In Mandy's case, for example, her male partner suggested that she was too “weak” to take her own medication—and thus incapable of having control over her own health—which could be perceived as the perpetrator asserting his assumed “masculinity” with male power expressed through tactical control (Stark, 2007). This reflects the traditional (i.e., patriarchal) notions of “masculinity” and “femininity,” where men/boys are seen as being in control, strong, decision-makers and women/girls as passive, fragile and dependent, and can be understood in terms of the relationship to gendered power relations that perpetuate and reinforce violence (Mays, 2006). Like Mandy, the abuse experienced by others such as Joanne and Kelly, mirrored the experiences of others whose abusers were also their “carers,” making them vulnerable to control (regulatory strategies are often disguised as “care” according to Stark, 2007) and abuse, including being denied funds for prescriptions which is a form of economic abuse (Women’s Aid, 2019). Additionally, the type of branding described by one practitioner is a form of extreme intimidation and a highly effective means of coercion and control, acting as a mark of the perpetrator's “ownership” of the victim-survivor visible to others (Stark, 2007). Male perpetrators deliberately denying their ex/partner (and/or their children) access to vital health intervention such as vaccinations, against the wishes of the victim-survivor/woman/mother, again may be seen as reflecting traditional (patriarchal) norms which serve to normalize the perception of male supremacy, and which often makes it more difficult to recognize controlling behaviors within an intimate relationship as abusive (Donovan & Hester, 2014; Women's Aid et al., 2021). After receiving specialist support for the abuse experienced, however, almost all participants recognized the intentionality of the abusive behaviors, understanding how the perpetrator had obtained control by use of a credible threat, which are key features of coercive control (Hamberger et al., 2017).

The (mis)use of chemicals, such as medication, in the ways described by victims-survivors in our study benefits the perpetrators by restricting the rights or independence of victims-survivors who may otherwise be able to resist their abuser with devastating effects on their health and well-being. Prescribed and nonprescribed medication (and other substances) are being used by perpetrators to psychologically, emotionally and physically harm, the key motivating factor being coercion, control and abuse of an intimate ex/partner. The strategies of perpetrators as outlined in this paper put victim-survivors at further risk of declining health and early mortality (Carthy & Holt, 2016) with no thought for the dignity or safety of victims, quite the opposite. And unlike the use of CR in health care or criminal justice settings, the use of chemical control in IPVA cannot be justified in any way, despite some perpetrators attempting to justify their behavior as benefiting the victim-survivor, such as in the cases of Anna and Claire, where they coerce the victim-survivor into taking medication that is in fact not needed.

## Conclusion

As shown, there are serious implications of perpetrators’ (mis)use of prescribed medication for the health of victims-survivors, not only any immediate pain or injury caused but the longer-term physical and mental health implications of not being able to take vital medication—that has often been specifically prescribed by a health professional—at either the right time, frequency or dosage, which could lead to worsening disease, hospitalization or even death. On the flipside there are the potential consequences of being administered with inappropriate or unnecessary medication. The experiences of victims-survivors such as Olivia demonstrate the potential for IPVA victims-survivors to develop opioid use disorder (OUD) as a direct result of abuse which then needs to be treated, with wider consequences including the cost to society of treating OUD with medication (which then has the potential to also be (mis)used by perpetrators where the perpetrator and victim-survivor are still in a relationship). As well as issues of potential addiction amongst victims-survivors who may have been coerced or forced into taking mediation that they do not actually need, which then need to be treated with substances that also come with a whole host of potentially serious side-effects including pain, insomnia and cognitive issues which can all affect daily life, there are also potential addiction issues amongst those perpetrators who may be (mis)using medicines that are not specifically prescribed for their own personal use.

Perpetrators can, and do, (mis)use prescribed or nonprescribed medication, sometimes in combination with alcohol and/or other substances, to coerce and control as part of an overall strategy to dominate the victim-survivor, harming their liberty and offending their human right to dignity, security and autonomy (Stark, 2007). Perpetrator tactics revealed in our study, including sabotage, intimidation and economic oppression, serve to reduce or erode a victim-survivor's right to health, to physical and mental integrity. As such the use of chemical control may be regarded as another “bar” of the invisible cage that victims-survivors of coercive control find themselves trapped within (Stark, 2007). Only by looking at those areas of coercion and control that are difficult, if not impossible, to evidence within the legal and medical frameworks, can we truly address the concerns of victims-survivors as they fight to survive in an unreality intended to rob them of their liberty (Williamson, 2010).

## Implications for Policy and Practice

The absence of a clear understanding of the importance of coercive control when making judgments about victims and perpetrators has serious implications for the efficacy of current approaches to domestic abuse (Robinson et al., 2018). Given the serious health implications of chemical control within IPVA for victims-survivors’ physical and mental health, there is a clear need for awareness and understanding amongst statutory and nonstatutory agencies working with victims-survivors, perpetrators and commissioning services, including the police, local authorities and the NHS about the nature of coercive control including how medication—and the intersection of illegal drugs and legal substances such as alcohol—can be weaponized within coercive controlling relationships and the harm caused. From a health perspective, practitioners such as GPs, pharmacists, community health workers, midwives and gerontological nurses must receive appropriate training regarding such perpetrator behaviors, and the impact on victims-survivors, to effectively support people experiencing this specific type of abuse. Primary care practitioners including GPs and pharmacists, should be supported to understand that perpetrators can, and do, use medication to abuse, in order to recognize potential red flags, for example, where a patient is either under or over requesting prescriptions for their medication and not improving as expected. As prescription medication is known to contribute to addiction (NICE, 2022) there are also implications for the prevention of drug-related harm which can be added to the overall cost (financial and otherwise) of domestic violence and abuse, in terms of physical, mental and reproductive health care needs of victims-survivors.

Specialist IPVA professionals interviewed as part of this study suggested that the use of chemical restraints amongst victims-survivors is much more of a problem than we currently know and that people accessing support for IPVA do not tend to disclose this type of behavior because often they do not recognize it as abuse, which was also supported by testimonies of victims-survivors many of whom said they did not necessarily recognize the abuse while still in the relationship. IPVA professionals may benefit from more specialized training to help survivors they support to recognize and disclose such abuse, perhaps by asking more or different questions, in order to more confidently respond to or mitigate its devastating impact on victims-survivors (and/or their children's) lives.

Coercive controlling abuse can be more devastating and salient to victims than physical violence (Stark, 2012) yet coercive control crimes remain far less likely to be prosecuted than domestic abuse crimes in general (Brennan & Myhill, 2022). Using chemical control in the ways illustrated in this paper can mean that perpetrators do not necessarily need to use physical violence to coerce and control the victim, which would be more likely to bring them to the attention of the authorities. Thus, criminal justice agencies may still be missing key opportunities for identifying “course of conduct” abuse meaning the coercive control offence is not being used to its full potential (Barlow et al., 2020). While it is a criminal offence to consume or possess any prescribed drugs not prescribed to you personally, the use of medication and other substances by perpetrators to coerce and control, including discrediting the survivor in child custody cases post-separation, must be recognized and understood as part of an on-going pattern of abuse which, taken together, serves to undermine and restrict the freedom of an individual victim with devastating on-going effects. This suggests a need for training of practitioners such as within Social Services and the criminal justice system to address the fact that it is extremely difficult for most survivors of IPVA to speak about this element of abuse and, even where the abuse may be relevant to alleged offending, disclosures may be limited initially, and survivors need to be given the space and opportunity to expand upon any abuse they mention (Swain Williams et al, 2022).

## Limitations

Despite efforts to reach as wide a demographic as possible our final sample was mostly White British, heterosexual women. We heard from only one male (hetero) victim-survivor and had no testimonies from gay or trans people. None of the participants were older than 54 years meaning we could not account for the experiences of older victims-survivors of IPVA, although these could be markedly different from those in younger age groups (McGarry et al., 2011). Demographic data for perpetrators may have also shed some light on who is doing what to whom and whether age might influence the particular chemicals and other substances used to “restrain” victim-survivors in the ways outlined in this paper. Thus, additional research on the sociocultural and relational contexts of the use of chemical restraints amongst all groups, including older, Black and minoritized, men and trans-IPVA survivors, is needed.

## Notes

1. ESRC Grant ES/M010090/1.

## References

[bibr1-10778012231197579] Alhusen J. L. Bloom T. Anderson J. Hughes R. B. (2020). Intimate partner violence, reproductive coercion, and unintended pregnancy in women with disabilities. Disability and Health Journal, 13(2), 100849. 10.1016/j.dhjo.2019.100849 31679950 PMC7096251

[bibr2-10778012231197579] Auerhahn K. Dermody Leonard E. (2000). Docile bodies–chemical restraints and the female inmate. Journal of Criminal Law and Criminology, 90(2), 599. 10.2307/1144231 11787499

[bibr3-10778012231197579] Bagwell-Gray M. E. Thaller J. Messing J. T. Durfee A. (2021). Women’s reproductive coercion and pregnancy avoidance: Associations with homicide risk, sexual violence, and religious abuse. Violence Against Women, 27(12-13), 2294–2312. 10.1177/10778012211005566 34165023

[bibr4-10778012231197579] Barlow C. Johnson K. Walklate S. Humphreys L. (2020). Putting coercive control into practice: Problems and possibilities. British Journal of Criminology, 60(1), 160–179. 10.1093/bjc/azz041

[bibr5-10778012231197579] Brennan I. Myhill A. (2022). Coercive control: Patterns in crimes, arrests and outcomes for a new domestic abuse offence. British Journal of Criminology, 62(2), 468–483. 10.1093/bjc/azab072

[bibr6-10778012231197579] Burman M. Brooks-Hay O. (2018). Aligning policy and law? The creation of a domestic abuse offence incorporating coercive control. Criminology & Criminal Justice, 18(1), 67–83. 10.1177/1748895817752223

[bibr7-10778012231197579] Callaghan J. E. M. Alexander J. H. Sixsmith J. Fellin L. C. (2018). Beyond “witnessing": Children's experiences of coercive control in domestic violence and abuse. Journal of Interpersonal Violence, 33(10), 1551–1581. 10.1177/0886260515618946 26663742

[bibr8-10778012231197579] Carthy N. Holt A. (2016). *Domestic abuse and older adults*. British Psychological Society, North East of England Branch Bulletin. https://research.tees.ac.uk/ws/portalfiles/portal/4039387/620583.pdf

[bibr9-10778012231197579] Crépault C. Kilty J. (2017). Mainstream media and the F-word: Documentary coherence and the exclusion of a feminist narrative in the fifth estate coverage of the Ashley Smith case. Canadian Journal of Law and Society/Revue Canadienne Droit Et Société, 32(2), 269–290. 10.1017/cls.2017.12

[bibr10-10778012231197579] Crespi T. D. (1990). Restraint and Seclusion with Institutionalized Adolescents. Adolescence, 25(100), 825–829. 2275438

[bibr11-10778012231197579] Donley M. Chan J. B. Webber L. S. (2012). Disability support workers’ knowledge and education needs about psychotropic medication. British Journal of Learning Disabilities, 40(4), 286–291. 10.1111/j.1468-3156.2011.00707.x

[bibr12-10778012231197579] Donovan C. Hester M. (2014). Domestic violence and sexuality: What’s love got to do with it? (1st ed.). Bristol University Press. 10.2307/j.ctt9qgzhk

[bibr13-10778012231197579] Edwards N. King J. Williams K. Hair S. (2020). Chemical restraint of adults with intellectual disability and challenging behaviour in Queensland, Australia: Views of statutory decision makers. Journal of Intellectual Disabilities, 24(2), 194–211. 10.1177/1744629518782064 29929418

[bibr14-10778012231197579] Fay K. Yee L. (2018). Reproductive coercion and women’s health. Journal of Midwifery and Women’s Health, 63(5), 518–525. 10.1111/jmwh.12885 30102016

[bibr15-10778012231197579] Gadd D. Henderson J. Radcliffe P. Stephens-Lewis D. Johnson A. Gilchrist G. (2019). The dynamics of domestic abuse and drug and alcohol dependency. British Journal of Criminology, 59(5), 1035–1053. 10.1093/bjc/azz011

[bibr16-10778012231197579] Gangoli G. Bates L. Hester M. (2019). What does justice mean to black and minority ethnic (BME) victims/survivors of gender-based violence? Journal of Ethnic and Migration Studies, 46(15), 3119–3135. 10.1080/1369183X.2019.1650010

[bibr17-10778012231197579] Gilchrist G. Dennis F. Radcliffe P. Henderson J. Howard L. M. Gadd D. (2019). The interplay between substance use and intimate partner violence perpetration: A meta-ethnography. International Journal of Drug Policy, 65, 8–23. 10.1016/j.drugpo.2018.12.009 30580114

[bibr18-10778012231197579] Grace K. T. Decker M. R. Alexander K. A. Campbell J. Miller E. Perrin N. Glass N. (2022). Reproductive coercion, intimate partner violence, and unintended pregnancy among Latina women. Journal of Interpersonal Violence, 37(3-4), 1604–1636. 10.1177/0886260520922363 32486886 PMC8162928

[bibr19-10778012231197579] Gulland A. (2016). GPs told to end chemical restraint of people with learning disabilities. British Medical Journal, 353, i3137. 10.1136/bmj.i3137 27260200

[bibr20-10778012231197579] Hague G. Thiara R. Mullender A. (2011). Disabled women, domestic violence and social care: The risk of isolation, vulnerability and neglect. British Journal of Social Work, 41(1), 148–165. 10.1093/bjsw/bcq057

[bibr21-10778012231197579] Hague G. M. Thiara R. Magowan P. Mullender A. (2008). Making the links: Disabled women and domestic violence. *Safe: The Domestic Abuse Quarterly, Summer, 1–5.* https://research-information.bris.ac.uk/en/publications/making-the-links-disabled-women-and-domestic-violence

[bibr22-10778012231197579] Hamberger L. K Larsen S. E. Lehrner A. (2017). Coercive control in intimate partner violence. Aggression and Violent Behavior, 37, 1–11. 10.1016/j.avb.2017.08.003

[bibr23-10778012231197579] Hardesty J. L. Ogolsky B. G. (2020). A socioecological perspective on intimate partner violence research: A decade in review. Journal of Marriage and Family, 82(1), 454–477. 10.1111/jomf.12664

[bibr24-10778012231197579] Hester M. Lilley S.-J. (2018). More than support to court: Rape victims and specialist sexual violence services. International Review of Victimology, 24(3), 313–328. 10.1177/0269758017742717 30111902 PMC6066863

[bibr25-10778012231197579] Hester M. Mulvihill N. Matolcsi A. Lanau Sanchez A. Walker S.-J. (2019). *The nature and prevalence of prostitution and sex work in England and Wales today*. University of Bristol. https://assets.publishing.service.gov.uk/government/uploads/system/uploads/attachment_data/file/842920/Prostitution_and_Sex_Work_Report.pdf

[bibr27-10778012231197579] HMIC (2014). *Everyone’s business: Improving the police response to domestic abuse*. ISBN: 978-1 78246-381-8. https://www.justiceinspectorates.gov.uk/hmicfrs/publications/improving-the-police-response-to-domestic-abuse/

[bibr28-10778012231197579] Kaskie B. P. Nattinger M. Potter A. (2015). Policies to protect persons with dementia in assisted living: Déjà vu all over again? The Gerontologist, 55(2), 199–209. 10.1093/geront/gnu179 26035596 PMC4542835

[bibr29-10778012231197579] Katz E. (2016). Beyond the physical incident model: How children living with domestic violence are harmed by and resist domestic violence. Child Abuse Review, 25(1), 46–59. 10.1002/car.2422

[bibr30-10778012231197579] Katz J. Poleshuck E. L. Beach B. Olin R. (2017). Reproductive coercion by male sexual partners: Associations with partner violence and college women’s sexual health. Journal of Interpersonal Violence, 32(21), 3301–3320. 10.1177/0886260515597441 26246119 PMC7144871

[bibr31-10778012231197579] Kelly L. Sharp N. Klein R. (2014). *Finding the costs of freedom. How women and children rebuild their lives after domestic violence* [Project Report]. Solace Women’s Aid. https://www.solacewomensaid.org/wp-content/uploads/2014/06/SWA-Finding-Costs-of-Freedom-Report.pdf

[bibr32-10778012231197579] Knight L. Hester M. (2016). Domestic violence and mental health in older adults. International Review of Psychiatry, 28(5), 464–474. 10.1080/09540261.2016.1215294 27564268

[bibr33-10778012231197579] Kovar C. L. (2018). Reproductive coercion: Baby, if you love me. The American Journal of Maternal-Child Nursing, 43(4), 213–217. 10.1097/NMC.0000000000000435 29958204

[bibr34-10778012231197579] Lazenbatt A. Devaney J. Gildea A. (2013). Older women living and coping with domestic violence. Community Practitioner, 86(2), 28–32. https://pubmed.ncbi.nlm.nih.gov/23469739/ 23469739

[bibr35-10778012231197579] Lee S. Gray R. Gournay K. Wright S. Parr A. M. Sayer J. (2003). Views of nursing staff on the use of physical restraint. Journal of Psychiatric and Mental Health Nursing, 10(4), 425–430. 10.1046/j.1365-2850.2003.00625.x 12887634

[bibr36-10778012231197579] Machado L. Gomes R. Xavier E. (2001). Report on elder abuse in Brazil. Candido Mendes University. 10.1590/1413-812320152111.23142016

[bibr37-10778012231197579] Macleod J. Cosgrove K. (1995). *We’re no exception: Male violence against women with disability* .

[bibr38-10778012231197579] Magowan P. (2004). The impact of disability on women's experiences of domestic abuse: An empirical study into disabled women’s experiences of, and responses to domestic abuse [Unpublished doctoral dissertation]. University of Nottingham.

[bibr39-10778012231197579] Marzullo L. R. (2014). Pharmacologic management of the agitated child. Pediatric Emergency Care, 30(4), 269–278. 10.1097/PEC.0000000000000112 24694885

[bibr40-10778012231197579] Matolcsi A. (2020). Unwanted sex with third parties in domestic abuse relationships and its impact on help-seeking and justice. Journal of Gender Based Violence, 4(1), 107–121. 10.1332/239868020X15791778136368

[bibr41-10778012231197579] Mays J. (2006). Feminist disability theory: Domestic violence against women with a *disability*. Disability and Society, 21(2), 147–158. 10.1080/09687590500498077

[bibr42-10778012231197579] McGarry J. Simpson C. Hinchliff-Smith K. (2011). The impact of domestic abuse for older women: A review of the literature. Health and Social Care Community, 19(1), 3–14. 10.1111/j.1365-2524.2010.00964.x 21040066

[bibr43-10778012231197579] McSherry B. Tellez J. J. (2016). Current challenges for the regulation of chemical restraint in health care settings. Journal of Law and Medicine, 24(1), 15–19. https://pubmed.ncbi.nlm.nih.gov/30136770/ 30136770

[bibr44-10778012231197579] Montreuil M. Thibeault C. McHarg L. Carnevale F. A. (2020). Moral experiences of crisis management in a child mental health setting: A participatory hermeneutic ethnographic study. Culture, Medicine & Psychiatry, 44, 80–109. 10.1007/s11013-019-09639-4 31218498

[bibr45-10778012231197579] Muir-Cochrane E. Grimmer K. Gerace A. Bastiampillai T. Oster C. (2020a). Prevalence of the use of chemical restraint in the management of challenging behaviours associated with adult mental health conditions: A meta-synthesis. Journal of Psychiatric Mental Health Nursing, 27(4), 425–445. 10.1111/jpm.12585 31867795

[bibr46-10778012231197579] Muir-Cochrane E. Oster C. Grimmer K. (2020b). Interrogating systematic review recommendations for effective chemical restraint. Journal of Evaluation in Clinical Practice, 26(6), 1768–1779. 10.1111/jep.13363 32059065

[bibr47-10778012231197579] National Institute for Health and Care Excellence (NICE). (2022). *Medicines associated with dependence or withdrawal symptoms: Safe prescribing and withdrawal management for adults* [NICE guideline. Published 20 April 2022]. www.nice.org.uk/guidance/ng215

[bibr48-10778012231197579] Nosek M. A. Howard C. A. Hughes R. B. (2001). The investigation of abuse and women with disabilities: Going beyond assumptions. Violence Against Women, 7(4), 477–499. 10.1177/10778010122182569

[bibr49-10778012231197579] O'Brien J. E. Ermentrout D. Li W. Dababnah S. Rizo C. F. Macy R. J. (2018). Measuring substance use among system-involved IPV survivors: A research note. Violence Against Women, 24(1), 101–119. 10.1177/1077801216675744 27881791

[bibr50-10778012231197579] Office for National Statistics (ONS). (2021). *Domestic abuse and the criminal justice system* (Nov 2021 dataset). https://www.ons.gov.uk/peoplepopulationandcommunity/crimeandjustice/datasets/domesticabuseandthecriminaljusticesystemappendixtables

[bibr51-10778012231197579] Office for National Statistics (ONS). (2022). *Partner abuse in detail* (Appendix tables). https://www.ons.gov.uk/peoplepopulationandcommunity/crimeandjustice/datasets/partnerabuseindetailappendixtables

[bibr52-10778012231197579] Plummer S.-B. Findley P. A. (2011). Women with disabilities’ experience with physical and sexual abuse: A review of the literature and implications for the field. Trauma, Violence, and Abuse, 13(1), 15–29. 10.1177/1524838011426014 22070987

[bibr53-10778012231197579] Preisz A. Preisz P. (2020). Restraint in paediatrics: A delicate balance. Journal of Paediatrics and Child Health, 55(10), 1165–1169. 10.1111/jpc.14607 31482670

[bibr54-10778012231197579] Ralph S. J. Espinet A. J. (2019). Use of antipsychotics and benzodiazepines for dementia: Time for action? What will be required before global de-prescribing? Dementia (London), 18(6), 2322–2339. 10.1177/1471301217746769 29271252

[bibr55-10778012231197579] Ritchie J. Lewis J. McNaughton Nicholls C. Ormston R. (2013). Qualitative research practice: A guide for social science students and researchers (2nd ed.). Sage. https://us.sagepub.com/en-us/nam/qualitative-research-practice/book237434

[bibr56-10778012231197579] Robinson A. Myhill A. Wire J. (2018). Practitioner (mis) understandings of coercive control in England and Wales. Criminology and Criminal Justice, 18(1), 29–49. 10.1177/1748895817728381

[bibr57-10778012231197579] Scott M. McKie L. Morton S. Seddon E. Wassof F. (2004). Older women and domestic violence in Scotland “…and for 39 years I got on with it”. Centre for Research on Families and Relationships and NHS Health Scotland.

[bibr58-10778012231197579] Social Care Institute for Excellence (SCIE) (2015). *At a glance 69: Safeguarding adults: Types and indicators of abuse*. https://www.scie.org.uk/safeguarding/adults/introduction/types-and-indicators-of-abuse

[bibr59-10778012231197579] Sorrentino A. (2004). Chemical restraints for the agitated, violent, or psychotic pediatric patient in the emergency department: Controversies and recommendations. Current Opinion in Paediatrics, 16(2), 201–205. 10.1097/00008480-200404000-00016 15021203

[bibr60-10778012231197579] Stark E. (2007). Coercive control: How men entrap women in personal life. Oxford University Press. https://psycnet.apa.org/record/2007-05264-000

[bibr61-10778012231197579] Stark E. (2012). *Re-presenting Battered Women: Coercive Control and the Defense of Liberty. Prepared for Violence Against Women: Complex Realities and New Issues in a Changing World*. Les Presses de l’Université du Quebec. https://www.stopvaw.org/uploads/evan_stark_article_final_100812.pdf

[bibr62-10778012231197579] Stark E. Hester M. (2019). Coercive control: Update and review. Violence Against Women, 25(1), 81–104. 10.1177/1077801218816191 30803427

[bibr63-10778012231197579] Stöckl H. Penhale B. (2014). Intimate partner violence and its associations with physical and mental health symptoms among older women in Germany. Journal of Interpersonal Violence, 30(17), 3089–3111. 10.1177/0886260514554427 25392386

[bibr64-10778012231197579] Swain Williams K. Storey A. Cooper S. L. (2022). *Double standard: Ending the unjust criminalisation of victims of violence against women and girls*. Centre for Women’s Justice. https://static1.squarespace.com/static/5aa98420f2e6b1ba0c874e42/t/6241a370051da468f5ba42d3/1648468856173/DS+FINAL+REPORT.pdf

[bibr66-10778012231197579] Thaller J. Messing J. T. (2016). Reproductive coercion by an intimate partner: Occurrence, associations, and interference with sexual health decision making. Health and Social Work, 41(1), 11–19. 10.1093/hsw/hlv083

[bibr67-10778012231197579] Walklate S. Fitz-Gibbon K. (2019). The criminalisation of coercive control: The power of law? International Journal for Crime, Justice and Social Democracy, 9(4), 94–108. 10.5204/IJCJSD.1829

[bibr68-10778012231197579] Webber L. S. McVilly K. R. Chan J. (2011). Restrictive interventions for people with a disability exhibiting challenging behaviours: Analysis of a population database. Journal of Applied Research in Intellectual Disabilities, 24(6), 495–507. 10.1111/j.1468-3148.2011.00635.x

[bibr69-10778012231197579] Williamson E . (2000). Domestic violence and health: the response of the medical profession. Policy Press.

[bibr70-10778012231197579] Williamson E. (2010). Living in the world of the domestic violence perpetrator: Negotiating the unreality of coercive control. Violence Against Women, 16(12), 1412–1423. 10.1177/1077801210389162 21164217

[bibr71-10778012231197579] Williamson E (2014). Heterosexual men as victims of domestic violence and abuse: prevalence, help-seeking and challenges to feminist theoretical frameworks. Understanding Gender Based Violence, 147–161.

[bibr72-10778012231197579] Women’s Aid. (2019). *The Domestic Abuse Report 2019: The Economics of Abuse*. Women’s Aid. http://www.womensaid.org.uk/evidence-hub-the-domestic-abuse-report-2019/

[bibr26-10778012231197579] Women’s Aid, Hester M. Walker S.-J. Williamson E. (2021). *Gendered experiences of justice and domestic abuse. Evidence for policy and practice*. https://www.womensaid.org.uk/wp-content/uploads/2021/07/FINAL-Gendered-experiences-WA-UoB-July-2021.pdf

[bibr73-10778012231197579] Wuest J. Merritt-Gray M. Ford-Gilboe M. Lent B. Varcoe C. Campbell J. C. (2008). Chronic pain in women survivors of intimate partner violence. The Journal of Pain, 9(11), 1049–1057. 10.1016/j.jpain.2008.06.009 18701353

[bibr74-10778012231197579] Wynn R. (2004). Psychiatric inpatients’ experiences with restraint. Journal of Forensic Psychiatry and Psychology, 15(1), 124–144. 10.1080/14789940410001655187

